# Standardization of Epidemiological Surveillance of Group A Streptococcal Pharyngitis^[Author-notes ofac251-FM1]^

**DOI:** 10.1093/ofid/ofac251

**Published:** 2022-09-15

**Authors:** Kate M Miller, Robert R Tanz, Stanford T Shulman, Jonathan R Carapetis, Thomas Cherian, Theresa Lamagni, Asha C Bowen, Janessa Pickering, Alma Fulurija, Hannah C Moore, Jeffrey W Cannon, Timothy C Barnett, Chris A Van Beneden, for the Strep A Vaccine Global Consortium (SAVAC) Burden of Disease Working Group, Jonathan Carapetis, Chris Van Beneden, David C Kaslow, Thomas Cherian, Theresa Lamagni, Mark Engel, Jeffrey Cannon, Hannah C Moore, Asha Bowen, Anna Seale, Gagandeep Kang, David Watkins, Sam Kariuki

**Affiliations:** Wesfarmers Centre for Vaccines and Infectious Diseases, Telethon Kids Institute, University of Western Australia, Perth, Australia; Northwestern University Feinberg School of Medicine and Ann and Robert H. Lurie Children’s Hospital of Chicago, Chicago, Illinois, USA; Northwestern University Feinberg School of Medicine and Ann and Robert H. Lurie Children’s Hospital of Chicago, Chicago, Illinois, USA; Wesfarmers Centre for Vaccines and Infectious Diseases, Telethon Kids Institute, University of Western Australia, Perth, Australia; Perth Children’s Hospital, Perth, Australia; MMGH Consulting, Geneva, Switzerland; United Kingdom Health Security Agency, London, United Kingdom; Wesfarmers Centre for Vaccines and Infectious Diseases, Telethon Kids Institute, University of Western Australia, Perth, Australia; Perth Children’s Hospital, Perth, Australia; Wesfarmers Centre for Vaccines and Infectious Diseases, Telethon Kids Institute, University of Western Australia, Perth, Australia; Wesfarmers Centre for Vaccines and Infectious Diseases, Telethon Kids Institute, University of Western Australia, Perth, Australia; Wesfarmers Centre for Vaccines and Infectious Diseases, Telethon Kids Institute, University of Western Australia, Perth, Australia; Wesfarmers Centre for Vaccines and Infectious Diseases, Telethon Kids Institute, University of Western Australia, Perth, Australia; Department of Global Health and Population, Harvard T. H. Chan School of Public Health, Boston, Massachusetts, USA; Wesfarmers Centre for Vaccines and Infectious Diseases, Telethon Kids Institute, University of Western Australia, Perth, Australia; CDC Foundation, Centers for Disease Control and Prevention, Atlanta, Georgia, USA

**Keywords:** epidemiology, infectious disease, pharyngitis, *Streptococcus pyogenes*, surveillance

## Abstract

Pharyngitis, more commonly known as sore throat, is caused by viral and/or bacterial infections. Group A *Streptococcus* (Strep A) is the most common bacterial cause of pharyngitis. Strep A pharyngitis is an acute, self-limiting disease but if undertreated can lead to suppurative complications, nonsuppurative poststreptococcal immune-mediated diseases, and toxigenic presentations. We present a standardized surveillance protocol, including case definitions for pharyngitis and Strep A pharyngitis, as well as case classifications that can be used to differentiate between suspected, probable, and confirmed cases. We discuss the current tests used to detect Strep A among persons with pharyngitis, including throat culture and point-of-care tests. The type of surveillance methodology depends on the resources available and the objectives of surveillance. Active surveillance and laboratory confirmation is the preferred method for case detection. Participant eligibility, the surveillance population and additional considerations for surveillance of pharyngitis are addressed, including baseline sampling, community engagement, frequency of screening and season. Finally, we discuss the core elements of case report forms for pharyngitis and provide guidance for the recording of severity and pain associated with the course of an episode.

## DISEASE CHARACTERISTICS

Group A *Streptococcus*, or *Streptococcus pyogenes* (Strep A), is the most common bacterial cause of pharyngitis (“strep throat”). Worldwide, approximately 615 million cases across all ages occur each year [1]. The palatine tonsils and surrounding oropharyngeal mucosa represents the major anatomical site responsible for maintaining the human reservoir of Strep A and for human-to-human transmission [2]. The peak age range of Strep A pharyngitis is between 5 and 15 years [3]. Strep A pharyngitis shows seasonal variation in temperate climates, with onset more common in winter and early spring [4–6].

Strep A pharyngitis commonly presents with the sudden onset of sore throat, pain in swallowing, and fever [7]. Physical signs and symptoms of Strep A pharyngitis include some or all of the following: sore throat, presence of pharyngeal erythema or exudate, palatal petechiae, tender anterior cervical lymph nodes, fever, and lack of common viral respiratory symptoms such as coryza, cough, laryngitis/hoarseness/croup, and conjunctivitis (Supplementary Appendix 1) [7]. Symptoms range from very mild to moderately severe illness.

Most pharyngitis episodes are benign and self-limiting, resolving within 1 week. However, a number of complications can occur including suppurative complications (cervical lymphadenitis, retropharyngeal abscess, peritonsillar cellulitis or abscess [quinsy], sinusitis, acute otitis media, and mastoiditis), nonsuppurative poststreptococcal immunologically mediated diseases (acute rheumatic fever [ARF], rheumatic heart disease, and acute poststreptococcal glomerulonephritis [ASPGN]), toxigenic presentations (scarlet fever, streptococcal toxic shock syndrome), and invasive diseases (eg, pneumonia) [8, 9].

Strep A pharyngitis is diagnosed by confirmation of the bacterium’s presence in the oropharynx, using throat culture or point-of-care (POC) tests, such as rapid antigen detection tests (RADTs) and nucleic acid amplification tests (NAATs). However, it can be difficult to distinguish between true Strep A pharyngitis and viral pharyngitis in the presence of Strep A carriage.

## OBJECTIVES OF SURVEILLANCE FOR STREP A PHARYNGITIS

An effective surveillance system for Strep A pharyngitis serves to (1) monitor trends in age- and sex-specific incidence (preferred) or period prevalence of symptomatic Strep A pharyngitis, accounting for season; (2) monitor trends in demographic and clinical characteristics of people with confirmed symptomatic Strep A pharyngitis; and (3) provide estimates of disease burden of Strep A pharyngitis estimates of disease burden.

Surveillance systems may also aim to determine and monitor age- and sex-specific incidence (preferred) or period prevalence of scarlet fever; the distribution of Strep A strains bearing selected genotypic or phenotypic features (eg, *emm* types, presence of vaccine antigens, and antimicrobial susceptibility); frequency of severe outcomes and complications of Strep A pharyngitis and scarlet fever; and age-specific burden of pharyngitis infections associated with other streptococci (ie, groups C and G), to measure the contribution of other β-hemolytic streptococci to overall pharyngitis burden and estimate the potential effect of Strep A common antigen vaccines on disease due to non-A streptococci.

Additional objectives that could be incorporated into routine surveillance or specialized (nonroutine) surveillance, vaccine trials, or research projects are provided in Supplementary Appendix 2.

## CASE DEFINITIONS AND CASE CLASSIFICATION

Standardized case definitions are important for obtaining accurate surveillance data, enabling comparisons of surveillance data across jurisdictions, and monitoring the impact of interventions. The definitions and methods in Table 1 may also be used as clinical endpoints for vaccine efficacy trials and postlicensure effectiveness studies. For most pharyngitis surveillance systems, 2 definitions should be used: pharyngitis (nonspecific) and Strep A pharyngitis.

**Table 1.** Case Definitions and Case Classification of Pharyngitis for Surveillance

Case Definitions
***Pharyngitis (nonspecific):*** Defined as an acute illness in a person with the complaint of sore throat or signs of pharyngitis (eg, erythema of pharynx and tonsils, patchy discrete exudate, and/or tender, enlarged anterior cervical nodes).
***Strep A pharyngitis:*** Defined as an acute clinical illness in a person (with the complaint of sore throat or clinical signs of pharyngitis) plus microbiological confirmation of Strep A in the oropharynx by a positive throat culture, RADT, or NAAT (Supplementary Appendix 3).
***Scarlet fever^a^:*** Defined as an illness characterized by a rash with diffuse erythema that blanches with pressure, with numerous small (1–2 mm) papular elevations plus evidence of antecedent or concomitant Strep A infection: elevated or rising ASO or anti-DNase B titers, OR isolation of Strep A from throat or skin sore culture, OR positive RADT or NAAT from throat swab (Supplementary Appendix 3).
**Case Classifications of Strep A Pharyngitis**
***Suspected cases:*** Defined as a sudden onset of sore throat or pain on swallowing in a person, and for which microbiologic tests for Strep A in the oropharynx have not been performed. Suspected case definitions are typically used to screen for infections that should be further evaluated to determine if the infection is a probable or confirmed case of Strep A pharyngitis.
***Probable cases:*** Defined as a sudden onset of sore throat or pain on swallowing in a person who is diagnosed, by professional medical personnel, using a clinical algorithm for Strep A pharyngitis that has been validated for the population under surveillance, and for which microbiological tests for Strep A in the oropharynx have not been performed.
***Confirmed cases:*** Defined as a suspected case in a person from whom group A *Streptococcus* (or *Streptococcus pyogenes*) is identified by throat culture or a reliable point-of-care diagnostic test (RADT or NAAT) from a throat swab.

Abbreviations: ASO, Antistreptolysin O; NAAT, nucleic acid amplification test; RADT, rapid antigen detection test.

a A clinical description of scarlet fever is provided in Supplementary Appendix 3.

### Notes about Case Classifications

Sudden onset is defined as onset occurring in the course of ≤24 hours. For suspected, probable, and confirmed case definitions, an acute case is an illness in a person who has not met the case definition of Strep A pharyngitis within the previous 30 days. In some specialized surveillance systems or research projects, where *emm* typing is performed on Strep A cultured from the throat of persons with pharyngitis, the exception to this 30-day rule is a confirmed case of Strep A–positive sore throat within 30 days of the first case and for which a different *emm* type is identified. For example, if M or *emm* typing capacity is available, a pharyngeal Strep A infection within 30 days of a previous infection may be considered a new case only if the typing result indicated a different *emm* type than the initial case.

### Other Definitions

For specialized studies, research projects, and measuring clinical endpoints in vaccine efficacy trials, definitions such as Strep A carriage, serologically confirmed Strep A pharyngitis, and other more nuanced definitions may be needed and are provided in Supplementary Appendix 4.

## MICROBIOLOGICAL TESTS USED TO DETECT STREP A AMONG PERSONS WITH PHARYNGITIS

Microbiological tests are necessary to confirm whether Strep A is present in the oropharynx, as clinical symptoms alone do not discriminate between Strep A and viral pharyngitis [10, 11]. Three microbiological diagnostic tests are currently available for detection of Strep A from a throat swab specimen and include bacterial culture and 2 rapid POC tests (RADTs and NAATs). The key features, advantages, and disadvantages of these diagnostic methods are described in Supplementary Appendix 5. The identification of Strep A is affected by the sensitivity of the test method; the tests used to detect Strep A in surveillance will therefore affect case ascertainment. Bacterial culture is currently considered the gold standard diagnostic test for Strep A; however, POC tests are an important option for surveillance studies in resource-poor settings or locations where microbiology laboratories are not available [12, 13].

Studies have demonstrated that the sensitivity of throat culture and RADTs is affected by clinical presentation (ie, spectrum effect/spectrum bias). Therefore, it is suggested that testing for Strep A be performed only if the clinical presentation suggests Strep A infection. Selectively using diagnostic testing for a subset of patients who present with a higher McIsaac score (greater pretest probability) is associated with greater test sensitivity [14] but will miss some patients with Strep A pharyngitis who have low McIsaac scores. Modification of this approach, with more liberal testing for Strep A to increase detection of Strep A pharyngitis, regardless of symptomology, may be indicated in communities or populations with high rates of ARF or ASPGN, or during local outbreaks of pharyngitis or invasive Strep A infections.

### Bacterial Culture

Cultures of throat swabs collected from a symptomatic person are commonly utilized in surveillance due to their high sensitivity. If sampling and plating techniques are conducted correctly, a single swab throat culture on a blood agar plate is 90%–95% sensitive for detecting Strep A pharyngitis [11, 15]. Cultures can also be used to detect and determine the frequency of other β-hemolytic streptococci including groups C and G. Culture is necessary to obtain the Strep A isolate if further characterization, such as *emm* typing, antibiotic susceptibility testing, or whole genome sequencing, is an objective of the surveillance program.

Culture of swabs is performed in a laboratory setting. Typically, throat swabs are inoculated onto blood agar plates; however, selective plates can be used to select for growth of Strep A [15]. Inoculated agar plates are initially incubated at 37°C for 18–24 hours, but incubation up to 48 hours may be necessary. The addition of 5%–10% carbon dioxide for incubation may enhance growth but is not essential. Following incubation, plates are inspected for β-hemolytic colonies to undergo subculture purification and confirmation with further biochemical tests including latex agglutination testing for Lancefield groups A, C, and G; bacitracin sensitivity; and pyrrolidonyl arylamidase testing. No biochemical test is 100% specific for *S pyogenes*, and therefore these tests are frequently performed in combination [16]. Purified colonies can be stored for further testing, with long-term storage between −70°C and −80°C in a suitable cryoprotectant medium such as in Todd-Hewitt glycerol broth or skim milk tryptone glucose glycerol broth (STGGB).

### Point-of-Care Tests

Point-of-care tests such as NAATs or RADTs are commercially available for oropharyngeal specimens. When locally validated, they are acceptable alternatives to culture in clinical practice due to their ease of use and ability to produce results rapidly. Point-of-care tests are benchmarked against bacterial culture to report sensitivity (percentage of culture-positive specimens that are detected by the POC test) and specificity (percentage of culture-negative specimens that are also negative by the POC test). Sensitivity and specificity of POC tests will differ depending on the manufacturer and should be taken into consideration when selecting tests for surveillance.

#### Nucleic acid amplification tests

NAATs are a molecular test for detecting Strep A DNA in pharyngeal swab specimens. NAATs are available in rapid (<15 minutes to 1 hour) and easy-to-use commercial formats. Recent studies have found that NAATs have equal or greater specificity than most RADTs and are a more sensitive (97.5%) POC test than RADTs [17, 18]. Therefore, negative NAATs do not require backup culture [19–21].

#### Rapid antigen detection tests

RADTs are used to identify the specific Strep A cell-wall antigen, the Lancefield group A carbohydrate. Depending on the manufacturer, these tests can be a latex agglutination assay, enzyme immunoassay, or optical enzyme immunoassay [22]. RADTs provide rapid results (<10 minutes). Due to the sensitivity (<90%) of RADTs, it is recommended that a throat culture or molecular test (eg, NAAT) be performed for Strep A in children and adolescents if the RADT is negative and clinical presentation suggests Strep A pharyngitis [23]. Current United States pharyngitis clinical guidelines state that children with symptoms of pharyngitis who test negative by RADT should have a backup throat culture performed to identify Strep A and initiate antibiotic therapy [7]. However, when the objective is to conduct *surveillance* for *emm* types or antibiotic resistance among Strep A isolates, pharyngitis surveillance programs can routinely use RADTs on all children with symptomatic pharyngitis and perform throat cultures on a representative sample of children with positive RADT specimens to obtain isolates for further characterization such as *emm* typing, antibiotic susceptibility testing, and whole genome sequencing.

### Other Methods

#### Antibody detection tests

Antibody detection tests measure the immune response to Strep A proteins in patient blood samples rather than the presence of the bacteria via throat culture, RADT, or NAAT. They are not typically used in routine pharyngitis surveillance but are necessary in research projects that aim to differentiate between people with acute Strep A pharyngitis and Strep A carriers with concurrent viral pharyngitis, or that aim to identify asymptomatic but immunologically significant Strep A pharyngeal infections. Further discussion of antibody detections tests is provided in Supplementary Appendix 6.

#### Clinical algorithms

In some resource-poor settings, bacterial culture and/or POC testing is not available or economically feasible [24]. Given that these same settings can be most at risk for poststreptococcal sequelae, syndromic pharyngitis surveillance when conducted consistently over time may be used to document disease burden and evaluate the impact of interventions. In lieu of a diagnostic test, investigators should use validated clinical algorithms (such as the Centor criteria [25], McIsaac score [26], and FeverPAIN score [27]) to guide clinical examination and diagnosis by assisting in differentiating typical bacterial symptoms (fever, presence of tonsillar exudate, swollen anterior cervical lymph nodes, and absence of cough) from typical viral features (cough, rhinorrhea, oral ulcers, conjunctivitis, and hoarseness of voice) [8]. The clinical presentation of childhood Strep A pharyngitis can vary between populations [28]. Accordingly, research has shown that the validity of clinical algorithms depends on the population in which they are conducted [24, 29].

## SPECIMEN COLLECTION FOR BACTERIAL THROAT CULTURE

### Equipment and Supplies

The following equipment and supplies are needed: (1) Gloves (need not be sterile); (2) Sterile swabs (calcium alginate, rayon, Dacron, or nylon materials) [30]; (3) Culture medium (eg, STGGB or room temperature–stablealternative); (4) Tongue depressor; (5) Flashlight; (6) Biohazard plastic bags, or clean plastic bags that can be labeled; (7) Transport container; and (8) Cooling bricks (if refrigerated storage is recommend for choice of culture medium).

### Methods of Sample Collection

Proper technique increases the yield of throat cultures in children. Persons collecting throat swabs should receive training in the following technique:

1. Verify the participant’s identity and label a sterile culture swab with the information requested by the protocol. This typically includes 2 patient identifiers (e.g., initials and surveillance visit number), date, and surveillance or site identity.
2. Put on gloves.
3. Position the child to face the brightest part of the room. If available, have 1 person steady the child’s head.
4. Shine a bright flashlight or penlight in the child’s mouth.
5. Use the other hand to remove the throat swab from its protective covering taking care to keep the tip sterile.
6. Ask the participant to open the mouth widely, protrude the tongue and say, “ahhh” or pant to elevate the uvula. Swabbing is best done under direct visualization and with the aid of a tongue depressor placed about three-quarters of the way to the posterior edge of the tongue to push the tongue down (inferiorly) firmly.
7. Rub the swab quickly but thoroughly over both tonsils (or tonsillar fossa) and the posterior pharyngeal wall of the pharynx using light pressure. Any exudate present should be swabbed. Other areas of the oral pharynx and mouth (eg, inside of cheeks) are not acceptable sites. Avoid contamination of the swab with saliva, the tongue, or the oral cavity.
8. Carefully store swab in culture medium if culture is not performed immediately (ie, place swabs in STGGB medium [31] and keep the swabs cold until freezing or plating).

### Storage and Handling

The following storage and handling should be taken. (1) Make sure the top is screwed on or pushed on firmly in place. (2) All specimens should be stored in sealed biohazard plastic bags or inside a biohazard-labeled sealed container: Store at the temperature required by culture medium. For example, room temperature storage is suitable for eSwabs (Copan, Italy), whereas refrigerated (in fridge) conditions are recommended for specimens stored in STGGB. (3) Sample collection documentation must be kept with specimens, but not in the same compartment in case of leakage.

### Documentation

The following documentation procedures should be taken. (1) Label all specimens: follow instructions on sticky label on tube/swab container; minimum information needed: unique participant ID number, date specimen collected, and type of specimen (eg, blood, wound swab); (2) A specimen transport log form should be used, consisting of: place, date and time of collection shipment and contents of shipment including participant ID numbers, specimen types and order of storage.

### Specimen Transfer

The following procedures should be taken for specimen transfer. (1) Place absorbent material in sealed biohazard bags with specimens in case of sample leakage; (2) Put into recommended portable transport container. For samples collected into storage medium with refrigeration recommended (ie, STGGB), store sealed bags in between ice cooler bricks; (3) Seal lid of portable container as instructed or with waterproof tape; (4) Label all containers clearly with: place, date, time of packing, and destination and biohazard sticker (if no sticker, write it in big letters using black marker); and (5) Make sure the courier knows what contents are, so they will not be left in a hot place and will be promptly delivered to the laboratory.

## TYPES OF SURVEILLANCE

The selection of surveillance strategies depends on specific epidemiologic and clinical characteristics of the disease outcome of interest, the overall surveillance objectives, surveillance location, services accessibility, and the resources available to conduct surveillance (see Supplementary Appendix 7 for key surveillance definitions). For example, in resource-poor settings, the resources required for active surveillance and laboratory confirmation may not be available, and case-finding activities may be inhibited. Given that those in resource-poor settings are often most at risk for post pharyngitis sequelae, surveillance is an important component of disease monitoring and control. Reliable burden estimates will inform the public health response to pharyngitis, advocate for vaccine use, and enable monitoring of the effect of interventions. Minimal and enhanced surveillance strategies for Strep A pharyngitis are described in Table 2 to provide guidance for those with limited resources and those with greater capacity, respectively.

**Table 2.** Surveillance Strategies for Strep A Pharyngitis

*Minimal Surveillance*
Llimited to passive surveillance of primary healthcare facilities. Based on clinical signs and symptoms or a diagnosis recorded in health facility databases, or microbiological data from laboratory databases. Settings include primary healthcare clinics such as outpatient clinics, doctors’ offices and hospitals, school-based clinics, and clinical laboratories. Participants seek medical care at healthcare or other relevant settings. If the provider or surveillance officer determines that the case definition for pharyngitis has been met, it is recorded in the EMR or a report provided to the surveillance system or local public health authorities. In the absence of access to microbiologic tests, diagnosis may rely on a clinical algorithm that has been validated for the specific population under surveillance. Surveillance staff implementing the clinical algorithm should be appropriately trained. Standard case report forms may be provided to the health facilities or laboratories for completion and submission to the surveillance program. This surveillance approach is appropriate when a minimum estimate of disease burden is considered adequate for surveillance purposes and the population at risk is well-characterized demographically [32].
*Enhanced Surveillance*
Includes prospective active case finding and laboratory confirmation among a large and well-defined population Active surveillance requires timely detection of new cases to ensure appropriate testing is conducted—throat culture or NAAT at the time of initial clinical assessment of symptomatic disease. This can be augmented by acute and convalescent serological testing in specialized surveillance systems or research projects. Participants are followed prospectively, ideally with frequent, regular contact, for a defined period using standard methods to collect demographics, clinical information, and microbiological testing to confirm Strep A cases. Settings include households, early childhood centers, schools and primary healthcare clinics. Clinical algorithms and predefined scores or symptoms/signs can be used to standardize the diagnostic testing approach for microbiological confirmation. Well-defined clinical practices and laboratory methods are established prior to surveillance and remain constant throughout the surveillance period. Audits should be performed biannually to assess the completeness of case ascertainment, accuracy, timeliness, and laboratory performance. Regular feedback of data/information is provided to healthcare workers and others involved in the surveillance process. This critical communication engages healthcare workers in the process and informs their clinical practice.

Abbreviations: EMR, electronic medical record; NAAT, nucleic acid amplification test.

A quality management plan should be written before the start of surveillance to establish and ensure the quality of processes, data, and documentation associated with surveillance activities. Moreover, all surveillance should be conducted in accordance with ethical guidelines (Supplementary Appendix 8).

## CASE ASCERTAINMENT AND SURVEILLANCE SETTINGS

For each data source, surveillance staff should (1) know the purpose of the data source and whether data have been routinely collected as part of patient care, mandatory collection of data under legal mandates, collected for research purposes, or other; (2) identify any legal mandates governing the operations of the data source that may affect the accessibility or quality of data from that source; and (3) describe the representative population for the data. Case ascertainment may be active or passive (Supplementary Appendix 9).

###  

#### Households

Active surveillance of households can identify persons with Strep A carriage and pharyngitis or sore throat who are unable to attend school, do not seek healthcare, or are unable to do so due to lack of time, financial constraints, or accessibility issues. Household surveillance provides the data required to determine the population at risk and calculate the overall disease burden. Population-based household surveillance reduces bias arising from inequalities in school and healthcare access but is time intensive, resource intensive, and costly. In communities with ubiquitous internet access, these challenges are being increasingly addressed by significant advances in the capacity to contact participants and collect surveillance data using digital technologies. The timing of survey implementation, if conducted in person or via telephone, must consider local and cultural schedules and customs to maximize the impact of the household surveys.

#### Early childhood centers and schools

Childcare centers, preschool, and primary schools offer a practical setting for disease and Strep A carriage surveillance, given that most surveillance programs are expected to include school-aged children. Childcare centers and schools are ideally placed to assess outbreaks of scarlet fever (frequency, size, and duration). Certain biases and sampling frameworks need to be considered within school settings to estimate the disease burden accurately. For example, surveying school attendees will lead to selection bias in a population with high levels of school absenteeism, resulting in an underestimate of disease burden. Factors associated with school nonattendance may include pharyngitis itself and conditions associated with the risk of pharyngitis such as poverty and/or general ill health. The bias should be acknowledged and school attendance rates cited. If possible, school nonattenders should be surveyed, although this is more difficult and costly. Sore throat clinics in the school setting can overestimate the incidence of Strep A pharyngitis when a sore throat complaint results in secondary gain (eg, time out of the classroom for clinical examination) or an environment of increased attention on sore throats leads to psychosomatic symptoms.

#### Primary healthcare

Active surveillance using primary healthcare clinics involves recruiting participants via their local primary healthcare clinic and requesting them to present to the clinic upon symptom development of a sore throat/pharyngitis. The symptoms should be sufficient to warrant a visit to the primary healthcare clinic (eg, Centor Score ≥2, McIsaac score ≥2). Predefined criteria to trigger healthcare access and regular symptom monitoring are key active surveillance strategies within primary healthcare settings. In addition, seasonal sampling of all subjects enrolled in healthcare-based studies should be considered for carriage surveillance to gain insight into baseline microbiology and future vaccine impact. Primary healthcare surveillance relies on engagement from surveillance staff, primary practitioners, and primary carers to maintain adequate retention rates, particularly for prospective longitudinal surveillance.

Passive surveillance using primary healthcare settings involves recording data on patients who present to primary healthcare clinics and represents an incomplete sample of children with pharyngitis or Strep A pharyngitis in the community, as it is commonly regarded as a transient and minor illness due to the often-mild symptomology of pharyngitis [33]. Furthermore, the data reported can be incomplete because not all physicians will conduct a diagnostic test on patients with a sore throat to confirm Strep A pharyngitis (ie, the propensity to seek microbiological confirmation will vary between physicians), making it difficult to interpret comparisons of incidence rates between primary healthcare clinics. To avoid underestimating disease burden among those who present to health services and to ensure a uniform testing approach, it is recommended that predefined criteria (eg, Centor Score ≥2) are established prior to the surveillance period to guide the testing protocol. Primary care data will be biased toward more severe cases and are typically not population-based, precluding the capacity to generate accurate disease rates, given that numerators but not corresponding denominators are collected.

Emergency department data or hospital admission data can be used for surveillance. While presentation to emergency departments for sore throat is common, only the most severe cases of pharyngitis, such as those with complications, will result in hospital admission. Therefore, emergency department surveillance may be useful for pharyngitis, but hospital inpatient surveillance is not recommended as it will result in a large underestimation of incidence. See Supplementary Appendix 10 for considerations for using administrative health databases in surveillance.

## SURVEILLANCE POPULATION

A surveillance protocol should clearly describe enrollment eligibility criteria. Most protocols would benefit from surveying children aged 3–15 years; however, age eligibility can vary between sites, depending on local needs and capacity. Children already receiving prophylactic antibiotics for any cause (eg, rheumatic heart disease, surgical procedures) should not be excluded from pharyngitis surveillance; however, the use of prophylactic antibiotics should be recorded. Unless specifically relevant to the surveillance aims, we also recommend including persons with underlying immunocompromising conditions or chronic diseases in pharyngitis surveillance. In vaccine efficacy trials, persons who are immunocompromised or on prophylactic antibiotics should be excluded from phase 1 and phase 2 trials as it may be difficult to interpret serologic data and culture results, impacting vaccine efficacy measurements.

The surveillance population includes all eligible at-risk people from which cases of Strep A pharyngitis are identified. This population, or denominator, must be well-characterized a priori to derive meaningful disease burden estimates. Without an accurate account of all people in the population who could potentially be evaluated for Strep A pharyngitis, disease estimates may be under- or overestimated [34, 35].

Some settings allow population-wide data on disease burden to be recorded and analyzed. Examples include household surveillance in a representative sample in a community or healthcare setting that serves the entire community. In these cases, the surveillance population would be defined as all eligible people who reside in the community. Data accuracy must be assured if government-derived census data are used to determine the community’s demographic profile, such as the number of people in relevant age categories.

In instances where select primary healthcare facilities serve a portion of a population residing in the geographical catchment area, healthcare utilization surveys can be used to estimate the denominator corresponding to the cases of interest, improving the accuracy of disease burden estimates and enabling rate calculations [36]. The denominator is the number of patients within the geographical catchment area who would be expected to attend that primary healthcare facility if signs and symptoms of Strep A pharyngitis develop. Cases not residing in the defined catchment area should be excluded.

When undertaking surveillance in a sample of schools and/or classrooms, the surveillance population is the number of children who agree, and have parental or guardian appropriate consent, to participate in surveillance. The results can be generalized to the entire community if schools and classes are randomized at the start of surveillance or appropriate demographic characteristics of participants can be weighed against the characteristics of the catchment population.

## SPECIAL CONSIDERATIONS FOR PHARYNGITIS SURVEILLANCE

### Administrative database review

Codes used to identify Strep A pharyngitis in electronic medical records (EMRs) are shown in Table 3.

**Table 3.** Specific Codes for Pharyngitis in Electronic Medical Record Databases

Type of Healthcare System	Pharyngitis Code
Primary healthcare system	
*International Classification of Primary Care*, version 2 (*ICPC-2*) system	R72 (strep throat and scarlet fever)
Read system	A340
SNOMED CT	43878008
Hospital data system	
*International Statistical Classification of Diseases and Related Health Problems, Tenth Revision* (*ICD-10*) [37]	J02.9 (acute pharyngitis unspecified) J02.0 (Strep A pharyngitis)

Abbreviations: CT, clinical terms; SNOMED, systematized nomenclature of medicine.

### Registers for scarlet fever

In some countries such as Austria, Poland, England, and Wales, scarlet fever is a notifiable disease. Scarlet fever registers play an essential role in identifying disease outbreaks, tracking disease spread, and identifying at-risk groups. Data can be used to inform disease control efforts, state decision-makers, and funding activities. They can also be used for research, providing the necessary information to monitor, control, and prevent scarlet fever in the community.

### Baseline sampling

Cross-sectional sampling within the first month of surveillance to gain baseline data can be useful for establishing the prevalence of asymptomatic Strep A throat carriage and determining the normal values for serum antistreptolysin O and antistreptococcal DNAse B titers within the surveillance population. However, carriage rates and baseline serology titers can vary by age and season. Baseline sampling can be used to establish a background Strep A antibody level/titer at the individual level to compare with serology results obtained following acute infection to detect a rise in levels. It is recommended that children with an acute sore throat on the day of swabbing or complaint of a sore throat in the week prior be excluded from any baseline sampling of Strep A carriage. Note that this is only necessary for some specialized surveillance systems and research studies such as those concerned with Strep A pharyngitis and postinfection sequalae, immunokinetics and vaccine efficacy studies.

### Sentinel surveillance at primary care sites

Establishing sentinel site surveillance for pharyngitis at primary care sites enables the systematic collection of oropharyngeal swabs from patients with pharyngitis. Swabs can then be submitted, cultured, and characterized at a centralized laboratory. Such sentinel surveillance can provide useful information on trends in etiology of pharyngitis and on strain types and antibiotic resistance patterns among pharyngitis infections due to Strep A.

### Community engagement

Community engagement during each surveillance step helps provide a considered approach to surveillance. Meaningful engagement can help ensure that the project is of value to the community and that community members have an opportunity to express their values and concerns and develop a degree of ownership. The time required to forge relationships between surveillance staff and communities should not be underestimated and must be built into the surveillance protocol.

The level of community engagement in the design, implementation, monitoring, and evaluation of surveillance will depend on the available resources and community capacity. Key stakeholders can include community members such as community leaders, teachers, and volunteers and staff from the local healthcare services including indigenous/community health workers, nurses, and general practitioners.

### Frequency

Untreated Strep A pharyngitis symptoms are usually self-limiting and resolve within a few days. Therefore, to detect new cases while Strep A can be recovered from a throat culture, we recommend that active surveillance for sore throat be conducted at least once every 2 weeks or once per week if resources allow. The schedule needed for passive surveillance will vary according to the pattern of case presentation at the surveillance site.

### Period of surveillance

Defining the surveillance duration depends on the availability of resources to support the surveillance system and the time needed to achieve the surveillance objectives. A minimum of 1 year is recommended due to the influence of seasonality (see below). Multiple years of surveillance are generally required to evaluate temporal trends and M or emm type distribution, or the impact of an intervention such as introducing a vaccine program.

### Season

If possible, surveillance staff should conduct surveillance across all seasons to capture the changes in disease occurrence over time. In areas where seasonality is well-described, limiting surveillance to months when most cases are likely to occur has efficiencies but will inflate incidence estimates and should not be used to extrapolate to annual incidence rates. Continuous surveillance is optimal but may not be possible due to school holidays, extended absences from school to tend to farms or other family or community duties, access to remote areas during wet seasons, and closure of communities for cultural reasons. Sampling outside of peak season can be useful for establishing asymptomatic Strep A carriage rates within the surveillance population and the circulating strain types.

### Measurement of disease burden

It is acceptable to count an infection in the same person as a new case each time an infection meets the case definition during the surveillance period, if 1 of the following situations also applies: (1) the *emm* type of Strep A causing the new pharyngitis case differs from the *emm* type causing the previous infection; or (2) at least 30 days have passed since the onset of the prior pharyngitis case.

When calculating cumulative incidence, the proportion of individuals in the population who experience a first episode of Strep A pharyngitis during a specified period, the numerator is affected children, not episodes, and the denominator is the population at risk at the beginning of the period. Period prevalence describes the proportion of the population under surveillance that had Strep A pharyngitis during a specified period which, due to the brief duration of acute Strep A pharyngitis, is comparable to cumulative incidence for the same period. Point prevalence is not recommended because Strep A pharyngitis has a brief duration and is seasonal.

“Prevalence of Strep A pharyngitis” is sometimes misused to refer to the proportion or percentage of all sore throat cases from which Strep A can be cultured. It is recommended that the term prevalence of positive tests be used for this purpose.

## DATA COLLECTION AND CASE REPORT FORMS

### Consent

Before initiating an assessment and collecting data or specimens, consent for participation in the surveillance program may need to be obtained based on the determination of an institutional review board. For children, consent needs to be obtained from their parent or legal guardian, and before examining, request permission (assent) from the child. Consent should be voluntary and based on sufficient information and an adequate understanding of the proposed surveillance program and the implications of participation. Flip charts and interpreters may help improve information delivery so that participants are clear about what they are consenting to. If consent is not obtained, do not proceed. For prospective active surveillance programs, each participant must be informed that participation in the project is voluntary and that they are free to withdraw, without justification, from the surveillance system at any time without consequences. Note that the age at which consent can and should be given by the child will vary between countries/jurisdictions. It is the responsibility of surveillance staff to confirm the requirements of local, regional, or national authorities. Informed consent may be obtained for surveillance, throat examination, photos of throat, administration of throat swabs, and storage of swabs for future use such as genetic sequencing and transcriptome analysis.

### Case Report Forms

Case report forms should be based on collecting only the information required to achieve the surveillance objectives. See Supplementary Appendix 11 for a list of recommended and optional variables for inclusion in all case report forms. Case report forms can be paper based but, increasingly, secure electronic data forms are used. Electronic case report forms offer several benefits such as early detection of cases and timely information flow, relatively inexpensive operating costs, and improved data quality (accuracy and data completeness) via imbedded validation checks.

General surveillance variables include unique identifier, date and time of first enrollment or specimen collection, and site where participant is seen such as setting, location, postcode, state/province/region, and country. Each encounter should also record a surveillance visit number/episode number if repeated episodes from the same person are included.

Key demographic variables include date of birth or, if date of birth not available, age (in days or months if <12 months and otherwise in years), sex, ethnic origin/race, residential postcode, state/province/region, and country.

Clinical and epidemiologic variables include signs and symptoms, duration, and pain related to sore throat, epidemiologic risk factors, and details on the treatment of the infection, including antibiotic use.

Some children, particularly older ones, will explicitly complain of throat pain. Others may complain only of pain when swallowing (odynophagia). To detect current cases of pharyngitis, we recommend that investigators ask: “Do you have a sore throat now (today)?” In some settings, it may be appropriate to ask this question differently and use other educational aids to make the question easier to understand.

### Course of An Episode: Severity And Pain

If the severity of pharyngitis is measured, surveillance staff should grade the severity using reproducible methods where possible. For example, the Brodsky grading scale [38] has acceptable intra- and interobserver reproducibility for grading the severity of pharyngitis according to the size of tonsils proportionate to their coverage of oropharyngeal width. Similarly, to measure pain level, it is recommended that investigators use a validated tool for the age group under surveillance. The Faces Pain Scale–Revised [39] is a valid and reliable pain scale for children aged ≥4 years and can be used in children without tonsils.

## Supplementary Material

ofac251_Supplementary_Data

## Supplementary Data

Supplementary materials are available at *Open Forum Infectious Diseases* online. Consisting of data provided by the authors to benefit the reader, the posted materials are not copyedited and are the sole responsibility of the authors, so questions or comments should be addressed to the corresponding author.
