# Supplementary material for: Standardization of Epidemiological Surveillance of Group A Streptococcal Pharyngitis
Source: Open Forum Infect Dis. 2022 Sep 15;9(Suppl 1):S5–S14. doi: 10.1093/ofid/ofac251 (PMC9474939; doi:10.1093/ofid/ofac251)
Supplement: ofac251_Supplementary_Data [file ofac251_supplementary_data.docx]

**Standardization of Epidemiological Surveillance of Group A Streptococcal Pharyngitis**

Supplementary Appendices

Table of Contents

[Appendix 1: Epidemiologic and Clinical Features Suggestive of Group A Streptococcal and Viral Pharyngitis 2](#_Toc112314408)

[Appendix 2: Additional Surveillance Objectives for Routine and Specialized (Non-Routine) Surveillance 3](#_Toc112314409)

[Appendix 3: Clinical Description of Scarlet Fever 4](#_Toc112314410)

[Appendix 4: Additional Case Definitions 5](#_Toc112314411)

[Appendix 5: Comparison of Laboratory Tests Used to Confirm Strep A Pharyngitis 6](#_Toc112314412)

[Appendix 6: Strep A Serology 8](#_Toc112314413)

[Appendix 7: Definitions of Key Surveillance Terms 10](#_Toc112314414)

[Appendix 8: Good Practice and Ethical Considerations 11](#_Toc112314415)

[Appendix 9: Comparisons of advantges and disadvantages of active and passive surveillance 12](#_Toc112314416)

[Appendix 10: Administrative Health Databases 13](#_Toc112314417)

[Appendix 11: Suggested Variable to be Used to Record Clinical and Epidemiological Examination Findings 14](#_Toc112314418)

[References 17](#_Toc112314419)

## Appendix 1: Epidemiologic and Clinical Features Suggestive of Group A Streptococcal and Viral Pharyngitis ^1^

| **Feature, by Suspected Etiologic Agent** |
| --- |
| *Group A streptococcal pharyngitis* |
| - Sudden onset of sore throat - Age 5–15 years - Fever - Headache - Nausea, vomiting, abdominal pain - Tonsillopharyngeal inflammation - Patchy tonsillopharyngeal exudates - Palatal petechiae - Anterior cervical adenitis (tender nodes) - Winter and early spring presentation - History of exposure to Strep A pharyngitis - Scarlatiniform rash |
| *Viral pharyngitis* |
| - Conjunctivitis - Coryza - Cough - Diarrhea - Hoarseness - Discrete ulcerative stomatitis - Viral exanthema |

## Appendix 2: Additional Surveillance Objectives for Routine and Specialized (Non-Routine) Surveillance

Additional objectives that could be incorporated into routine surveillance:

- Develop and/or validate with concomitant laboratory confirmation clinical algorithms (specific to local epidemiology) to estimate Strep A pharyngitis incidence
- Validate emerging microbiology and point-of-care tests for the diagnosis of Strep A pharyngitis
- Obtain data inputs to quantify other measures of disease burden, including costs (e.g., primary caretaker time costs, and costs of diagnostics, treatment regimens, clinic or hospital stays), and impact of potential interventions, including vaccines
- Monitor rates of scarlet fever over time to identify potential outbreaks and trigger rapid public health response
- Establish passive surveillance for acute disease sequelae (cervical lymphadenitis, retropharyngeal abscess, peritonsillar cellulitis or abscess [quinsy], sinusitis, acute otitis media, and mastoiditis) of acute Strep A pharyngitis

Additional objectives that could be incorporated into specialized (non-routine) surveillance, vaccine trials, or research projects:

- Estimate incidence of serologically confirmed Strep A pharyngitis
- Estimate frequency (incidence or prevalence) of the continuum of clinical scenarios associated with Strep A in the pharynx: symptomatic Strep A pharyngitis, asymptomatic Strep A pharyngitis (with immune response), persistence of asymptomatic carriage of Strep A following resolution of infection, and prevalence of asymptomatic Strep A carriage (asymptomatic persistent carriage following acquisition and infection)
- Understand transmission dynamics of Strep A pharyngitis between individuals and the roles of symptomatic infection, asymptomatic pharyngitis, and carriage and their contribution (if any) to invasive Strep A disease
- Evaluate the effectiveness or impact of clinical case management
- Measure adherence to guidelines for treatment of Strep A pharyngitis and treatment failures

## Appendix 3: Clinical Description of Scarlet Fever

The initial symptoms of scarlet fever typically include sore throat, headache, fever, nausea, and vomiting. After 12–48 hours, the characteristic rash develops, giving a ‘sandpaper’ quality to the skin. It usually starts on the chest and abdomen. The rash does not typically appear on the face, although the face may appear flushed, but with circumoral pallor. Subsequently, the rash expands rapidly to cover the extremities; the palms and soles are usually spared. The rash is most marked in the skin folds of the inguinal, axillary, antecubital, and abdominal areas and about pressure points. It often exhibits a linear petechial character in the antecubital fossae and axillary folds, known as Pastia's lines. On lighter skin, the rash appears pink or red. On darker skin, it can be more difficult to see but can be felt on physical examination. Ultimately, the rash desquamates as the illness resolves over several days. Scarlet fever occurs predominantly in children and is less common in adults.

The rash only occurs in people who have had prior exposure to *S pyogenes* and results from a delayed-type skin reactivity to pyrogenic exotoxin produced by the bacteria. Therefore, a positive test for Strep A is required to confirm the diagnosis. When there is diagnostic uncertainty a positive Strep A test can be used to rule out measles and other viral exanthems, and Kawasaki Disease, which can sometimes appear similar. In children with scarlet fever who appear very unwell, especially if there are signs suggestive of sepsis, consideration should be given to whether the diagnosis is streptococcal toxic shock syndrome, in which case the diagnostic workup should also include blood cultures.

**Figure: Scarlet fever. Distinctive features include flushed cheeks with circumoral pallor, and fine pink/red rash with a sandpaper-like texture, typically first appearing on the trunk.**

**
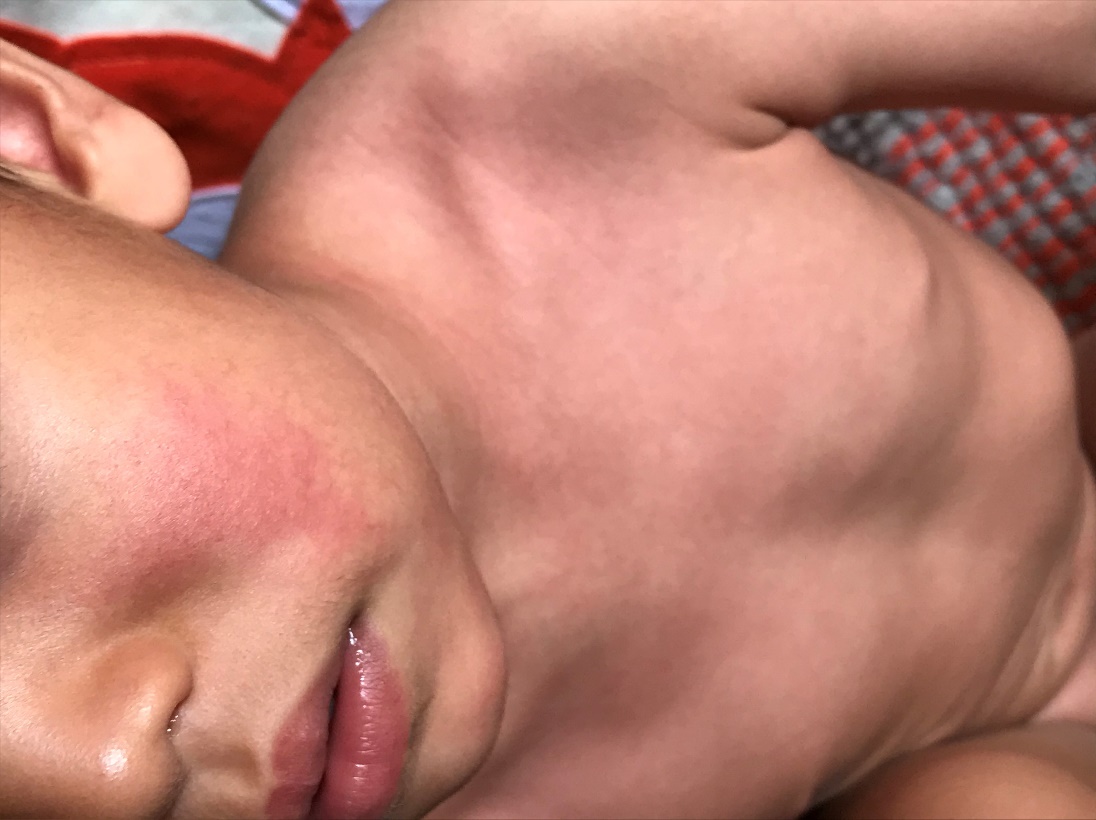
**

## Appendix 4: Additional Case Definitions

*Strep A throat carriage*

A case of Strep A throat carriage is defined as the identification (using bacterial culture or a validated point-of-care test) of Strep A in the throat of a person with no clinical symptoms of acute pharyngitis.

*Serologically confirmed Strep A pharyngitis*

A case of serologically confirmed Strep A pharyngitis is defined as Strep A pharyngitis (see above) in a person who has a positive antibody response to Strep A infection. Currently, just two titers are measured in clinical practice: ASO (anti-streptolysin O) and ADB (anti-deoxyribonuclease B),^[[1]](#footnote-1)^ defined as either:

- A two-fold or greater rise in titer in specimens collected at least two weeks apart (and preferably four weeks apart), with the first sample taken within one week of symptom onset.

OR

- A single sample taken at least two weeks after the onset of a sore throat that is above the upper limit of normal.

*Asymptomatic but immunologically significant Strep A pharyngitis*

A case of asymptomatic Strep A pharyngitis is defined as an infection in a person who has no clinical symptoms of acute pharyngitis but from whom Strep A is cultured from a throat swab and a serological response is documented.

Characterization of asymptomatic Strep A pharyngitis with an immune response can be useful for understanding the true burden of Strep A infections (as opposed to carriage) that may lead to post-streptococcal sequelae such as ARF.^2^

*Persistent asymptomatic throat carriage of Strep A following pharyngitis*

A case of persistent asymptomatic Strep A throat carriage following Strep A pharyngitis is defined as the confirmed continued presence^[[2]](#footnote-2)^ of Strep A in the throat, identified by throat culture or validated point of care test^[[3]](#footnote-3)^, in a person who had an asymptomatic pharyngitis infection confirmed by documented acute immune response. In this scenario, the same strain (e.g., same *emm* type) is cultured after the acute immune response has resolved.

*Asymptomatic acquisition and carriage of Strep A in the throat without an immune response*

Documented Strep A carriage without an immune response reflects the acquisition of Strep A in the throat in a person with no clinical symptoms of acute pharyngitis and for whom the appropriate serology tests are performed and are negative.

## Appendix 5: Comparison of Laboratory Tests Used to Confirm Strep A Pharyngitis

| Key Advantages | Key Disadvantages |
| --- | --- |
| *Throat culture* |  |
| - High sensitivity - High specificity - Most widely used in cohort studies - Allows storage of bacterial strains isolated, and genomic and/or phenotypic analysis (including antimicrobial susceptibility) - Allows concurrent identification of other bacterial species (e.g., groups C and G strep) | - Requires access to a laboratory and experienced microbiologist/clinical laboratory staff to grow and accurately identify Strep A - Labor intensive - Can be costly - Can delay results (1–5 days) - False negatives may occur if child is treated with antibiotics prior to culture collection - Does not distinguish between acute infection and Strep A carriage |
| *Rapid antigen diagnostic test (RADTs)* |  |
| - High specificity - Ease of use (requires minimum training to operate) - Low cost - Rapid turnaround time (<10 min), allowing for result and treatment in same visit as consultation - Can be used in low–middle income countries and/or regions that do not have the required laboratory infrastructure - Better access to testing, particularly for those in rural and remote sectors who may have limited or no access to clinical laboratories or pathology collection centers | - Due to varying sensitivities, may not be suitable in areas where acute rheumatic fever rates are high - Negative results must be confirmed by culture - Lower sensitivity than culture or NAAT - Does not distinguish between acute infection and Strep A carriage - Genomic analyses cannot be performed |
| *Nucleic acid amplification test (NAATs)* |  |
| - Likely higher sensitivity than throat culture and most RADTs - Culture confirmation not required - Can be used in a variety of settings - Rapid turnaround time | - Requires desktop analyzer and computer - Equipment is costly - Does not distinguish between acute infection and Strep A carriage - Genomic analyses cannot be performed |
| *Serological tests (not for diagnosis of acute infection)* |  |
| - Evidence of prior Strep A exposure through antibody tests: ASO (anti-streptolysin titers) and ADB (anti-DNase B) - Can differentiate between bacterial infection and colonization. - Can be used to confirm *bona fide* Strep A pharyngitis | - Cannot be used to identify the presence of a current bacterial infection at the time of presentation - Background data on community- and age-specific antibody levels required to appropriately interpret results. - Requires multiple testing - Not widely available in resource-poor settings - Requires intensive quality control - Costly - Genomic analyses cannot be performed |
| *Clinical algorithms (useful for screening in high resource settings and for probable diagnosis in limited-resource settings)* |  |
| - Can be used to screen patients for Strep A evaluation and inclusion in studies - Can be used in resource-poor countries - Lowest cost - Requires no equipment or specialized staff - Ease of use (requires minimum training to follow) - Can be adapted to surveillance population | - Clinical features can overlap other infections (e.g., viral pharyngitis) - Not generalizable and must be validated within the population under surveillance - Potential for false positives or negatives among patients with atypical clinical features - Needs periodic clinical audits to ensure that application of algorithms is uniform across the surveillance period |

## Appendix 6: Strep A Serology

Clinically, patients with signs and symptoms of Strep A pharyngitis and a positive test for Strep A are considered acutely infected with *S. pyogenes*. However, persons with viral pharyngitis may also carry Strep A in their throats without having Strep A pharyngitis. Antibody detection tests can be used to differentiate between people with an acute Strep A pharyngitis and Strep A carriers with concurrent viral pharyngitis. Antibody detection tests are not recommended for the diagnosis of acute pharyngitis in a symptomatic person (as they may result in false negatives) or useful for routine surveillance.

The time and expense of serologic testing used to confirm Strep A pharyngitis is best reserved for confirmation of Strep A infection in research studies and patients under evaluation for acute rheumatic fever (ARF) and acute post-streptococcal glomerulonephritis (APSGN). For example, antibody detection tests are necessary to detect asymptomatic but immunologically significant Strep A pharyngitis (as opposed to carriage), which have been reported in one study to represent 65% of new pharyngeal Strep A acquisitions.^2^ This aids our understanding of the true burden of Strep A infections. These asymptomatic but immunologically significant infections may lead to post-streptococcal sequelae such as ARF and APSGN.^2^

Serological testing is also important for vaccine trials and can be useful for informing Strep A immunogenicity to vaccine antigens and studying the kinetics of the Strep A antibody response in longitudinal cohorts.

Recent technological advances have seen the development of serological assays from dried blood spot (DBS) as a quick, cost-effective, and participant-friendly method for sero-epidemiology.^3,4^ DBS technology has been validated for use in anti-streptolysin O (ASO) titer detection.^5^

Traditional serological testing based on ASO and ADB titres may not always be reliable however newer tests based on multiplex technology have shown to be able to reliably measure antibodies to several Strep A proteins (ASO, ADB, SLO, SpyCEP, SpyAD, ScpA and SpnA) from a single low volume sample.^6,7^ The use of multiplex assays for Strep A serology will better inform the repertoire of anti-Strep A antibody responses in acute infection and increase reliability of testing where a rise in ASO or ADB is not observed (a rise may be seen in response to an alternative Strep A antigen). However, the associated costs may be a limiting factor in use of the assay

*Interpretation of results*

Interpretation of Strep A serology results can be difficult in communities with high incidence or prevalence of skin or upper respiratory Strep A infections^8^. In these settings, a negative antibody test helps exclude a recent infection, but a positive test does not necessarily indicate an infection in the past few weeks. Wherever possible, titers should be interpreted by comparing acute and convalescent samples and demonstrating a titer rise between these two time-points. Acute serum should be collected as soon as possible after presentation, and convalescent samples should align with the peak antibody titers (ASO and ADB) to optimize sensitivity. Because the timing of the rise for ASO and ADB differ slightly at 3–5 weeks and 6–8 weeks, respectively (although this has been shown to vary between individuals), it is recommended that convalescent samples be conducted between 4 and 6 weeks to best capture the rise across both titers. A 4-fold increase in titer from acute to convalescent (taken at least 2 weeks apart and preferably 4–6 weeks apart) is considered the gold standard, however a 2-fold increase is considered acceptable.

An upper limit of normal (ULN) cut-off (80^th^ percentile) can be used in place of the gold standard four-fold rise in titer when paired sera are not available. Ideally, age stratified ULN values for serum ASO and ADB titers will be available for a subset of healthy individuals, without recent Strep A infection, and drawn from the surveillance population of interest. Local data, where available, should be used to determine threshold titers as values can differ between and within countries based on variables such as ethnicity, geography, and socioeconomic status. However, developing local population ULN values is not always possible due to logistics, cost, or surveillance occurring in regions where streptococcal infections are endemic, and it is difficult to identify local children without a recent infection.

Recommended upper limits of normal for anti-streptolysin O and anti- DNase B titers, in the absence of appropriate local population data.

| **Age group (years)** | **Upper limit of normal (international units/mL)** | |
| --- | --- | --- |
|  | **ASO titer** | **Anti-DNase B titer** |
| 2-4 | 160 | 240 |
| 5-9 | 240 | 320-640 |
| 10-12 | 320 | 480-640 |
| >12 | 400 | 200 |

From: Kaplan EL et al, Pediatrics 1998; 101: 86-8; Gray GC et al. J Clin Epidemiol 1993; 46: 1181-5; and Karmarkar MG et al, Indian J Med Res. 2004;119 Suppl:26-8.

## Appendix 7: Definitions of Key Surveillance Terms

| **Syndromic surveillance** | Syndromic surveillance refers to the use of a clinical syndrome (a constellation of symptoms and signs) as the case definition for detection of suscept cases. Syndromic surveillance can be used for initial case detection, but laboratory confirmation should occur to increase the accuracy of the system.^9^ |
| --- | --- |
| **Active surveillance** | Active case detection means that designated public health surveillance staff are directly involved in detecting cases.^9^ |
| **Passive surveillance** | Passive case detection means that health facility staff detect and report cases to the public health system.^9^ |
| **Facility-based surveillance** | Facility-based surveillance is based on ascertainment of cases in persons who seek care at health facilities, including outpatient clinics, doctors’ offices, hospitals and emergency departments.^9^ |
| **Sentinel-site surveillance** | Sentinel-site surveillance refers to a system that captures cases at one or more specialized sites, such as hospitals, clinics, schools or pharmacies.^9^ |
| **Community-based surveillance** | Community-based surveillance is the systematic detection and reporting of events of public health significance within a community-by-community members. Community-based surveillance enables earlier detection of the disease of interest and captures illnesses in persons who do not seek care in a hospital.^10^ |
| **Population-based surveillance** | Population-based surveillance attempts to capture all cases in a well-defined catchment population (for example, the entire population of a country). |
| **Healthcare utilization surveys** | Healthcare utilization surveys seek to characterize the health care-seeking behavior of ill persons by describing where ill persons sought health care for their illnesses, and soliciting reasons for not seeking health care.^11^ |
| **Unique identifier** | Unique identifiers are unique numbers or numbers and letter combinations that are allocated to a specific individual person. |

## Appendix 8: Good Practice and Ethical Considerations

**Monitoring/Audit**

A systematic and independent audit of surveillance systems should be undertaken to ensure that surveillance and surveillance-related activities were conducted following the relevant surveillance protocol, SOPs, ethical guidelines, and regulatory requirement(s) established by local public health. Existing surveillance review tools can be modified to guide the investigation (e.g., WHO’s ‘[Tools for a surveillance review: Vaccine Preventable Diseases Surveillance Standards](https://www.who.int/publications/m/item/vaccine-preventable-diseases-surveillance-standards-annex1)’). Surveillance as part of a clinical study should adhere to the International Council for Harmonisation (ICH) Guidelines for Good Clinical Practice.^12^

**Quality control and quality assurance**

A quality management plan should be written before the start of surveillance to establish and ensure the quality of processes, data, and documentation associated with surveillance activities. It encompasses both quality control (QC) and quality assurance (QA) activities.

Surveillance systems should develop SOPs to ensure confidentiality for all cases, ensure that clinical specimens and bacterial isolates obtained are not compromised by human and processing errors, validate data integrity, and maintain multiple layers of security. A SOP will ideally detail:

- Data storage. Including participants’ unique surveillance ID numbers in each respective dataset enables linkage to other datasets, such as hospital admissions, facilitating the capture of complications and ensuring that all personal identifying information is removed from research/surveillance datasets.
- Data evaluation for protocol compliance and source document accuracy.
- Document review (e.g., specimen tracking logs, questionnaires), who is responsible, and frequency.
- Who the responsible person is for addressing QA issues (correcting procedures that do not comply with the surveillance protocol) and QC issues (correcting errors in data entry).
- Staff training activities and processes for documenting surveillance staff training.
- Maintenance and strict adherence to surveillance delegation log (list of staff involved in the surveillance and their duties/roles).
- Clinical and laboratory SOPs and accreditation.
- Regular audits of surveillance data to ensure accuracy and completion.
- System for periodic and refresher training for surveillance team.

**Ethics of surveillance**

The global network of WHO Collaborating Centres for Bioethics in collaboration with the U.S. Centers for Disease Control and Prevention developed ethical guidelines for public health surveillance, including common good, respect for persons, and good governance. The guidelines cover the (i) broad responsibility for undertaking surveillance and subjecting it to ethical scrutiny; (ii) obligation for ensuring appropriate protection and rights; (iii) considerations in making decisions about how to communicate and share surveillance data. The guidelines are available at <https://apps.who.int>. Countries should implement these guidelines and monitor them regularly. As appropriate, surveillance protocols should adhere to existing country-specific ethical guidelines.

## Appendix 9: Comparisons of Advantages and Disadvantages of Active and Passive Surveillance

| **Advantages** | **Disadvantages** |
| --- | --- |
| ***Active surveillance*** |  |
| - Sensitive system that facilitates early detection of new cases, contributing to prevention of post-infection sequalae - Higher case ascertainment rate - More accurate identification of cases - Ability to verify information in the case of missing data or suspected data entry errors - Data collected can be comprehensive and specific to the surveillance objectives - Can evaluate the quality and effectiveness of case-finding process, thus minimizing selection bias - Allows real-time analysis and ability to respond/modify approach to surveillance and care - Can promote disease awareness and good health practices | - Can be costly and resource-intensive - Requires dedicated surveillance staff and/or extensive training and upskilling - Can be demanding on surveillance sites - Barriers to accessing communities (e.g., distance/cultural barriers) |
| ***Passive surveillance*** |  |
| - Can be conducted retrospectively - Requires fewer resources than active surveillance - Can support real-time reporting | - Responsibility for reporting new cases lies with the healthcare workers/laboratory staff; thus, it can be difficult to ensure consistency of reporting by healthcare providers - Difficulties caused by lack of standardization in terms of case definitions and coding - Tends to under-report disease - Often difficult to confirm data recording or entry errors retrospectively - Commonly associated with incompleteness of data recording or of microbiological studies - Vulnerable to bias due to differences in physicians’ inclination to perform microbiological confirmation |

## Appendix 10: Administrative Health Databases

Administrative data from laboratory datasets and electronic medical records (EMRs) from primary healthcare and emergency departments covering whole communities can provide a timely and cost-effective surveillance option.

An important consideration when using EMRs to calculate disease estimates in a population is that the data are collected and coded as part of service delivery rather than for surveillance purposes. As such, EMRs are often prone to missing data on key fields and require the conversion of unstructured/narrative text, which can be resource-intensive and subjective. For EMRs that include or rely on free text, new methods in machine learning or deep learning could improve case identification.^13,14^ Data from EMRs are limited to patients who attend health services and are subject to variance in physician’s propensity to seek microbiological confirmation, which may be subject to bias (e.g., more severe infections, more clinically ambiguous, one not responding to treatment) and underestimate disease incidence. However, an advantage to administrative data is that, in well-established systems, data are collected systematically, well-structured and are often population-based. EMRs can form the basis of enhanced surveillance by using an additional data collection form to augment routinely collected data.

Routinely collected clinic data may be insufficient for evaluating potential cases against the full criteria required to meet surveillance case definitions, especially when microbiological testing is not routinely conducted or recorded. Further, the data may be insufficient for addressing other surveillance objectives, such as variant typing and antimicrobial susceptibility testing.

## Appendix 11: Suggested Variable to be Used to Record Clinical and Epidemiological Examination Findings

| Category of variables | Required variables | Optional variables |
| --- | --- | --- |
| **General** | - Participant unique ID number** - Date of enrolment | - Date of illness onset - Duration of symptoms |
| **Demographics** | - Age (in months if <1 year; otherwise in years) - Sex | - Date of birth - Race/ethnicity - Residential address |
| **Physical signs on clinical examination suggestive of Strep A as etiologic agent** |  | - Tonsillar erythema - Tonsillar swelling (graded 1–4 using tonsillar hypertrophy grading scale (Brodsky Grading Scale^15^, a standardized and reproducible measurement often used in pediatrics) - Tonsillar exudates - Pharyngeal erythema - Pharyngeal swelling - Pharyngeal exudates - Absence of cough - Current temperature (⁰C) - Palatal petechiae - Tender anterior cervical nodes - Anterior cervical node >1.5 cm in diameter - Scarlatiniform rash |
| **Physical signs on clinical examination suggestive of viral etiology** |  | - Rhinorrhoea (runny nose) - Cough - Conjunctivitis - Discrete oral ulcers - Anterior stomatitis - Other rash (describe) |
| **Epidemiologic risk factors** |  | - Age 3–15 years - Exposure history: pharyngitis among family or household members, classmates, or other close contacts of the child - Household crowding:   - number of household members   - number of bedrooms in household - Attendance in congregate setting, such as school, daycare (crowding increases risk of spread) - Season (winter or early spring in temperate climate) |
| **Clinical prediction rules score** |  | - Centor Criteria score; Modified McIsaac score; FeverPAIN score |
| **Prior treatment** | - Receipt of antibiotics in the past week | - Antibiotic prescribed at current surveillance visit - Antibiotic dose and frequency - Duration (in days) of antibiotic use - Analgesia/antipyretic use |
| **Diagnostic test** | - Diagnostic test used (culture, NAAT, RADT) - Date - Results | - Consent - Time of diagnostic test |
| **Serology** | If conducted:   - Date - Results | - Consent - Dried blood spot/blood draw - Time of test - Results |
| **Microbiology** | - Participant unique ID number** - Specimen unique IDǂ - Specimen collection date - Date and hour plate is inoculated - Date and hour plate is placed in incubator - Date(s) and hour(s) plate is read - Name of reporting laboratory - Laboratory ID (if present) - Episode number (if repeated episodes from the same person are included) - Group A *Streptococcus* or *Streptococcus* *pyogenes* identified: yes/no - Type of test (i.e., culture, RADT, NAAT, serology) | - β-hemolytic *Streptococcus* identified: yes/no   - If yes, group identified (choose one): A, B, C, G or other - Anti-streptolysin O (ASO) and anti-DNase B antibodies and SpnA - Date of notification of result to participant and/or doctor   - Storage/transport identification number - Place/site of transfer of isolate for additional testing - Further testing ordered (e.g., *emm* typing, anti-streptococcal antibody titers, speciation of large-colony β-hemolytic *Streptococcus*, etc.) - Whole-genome sequencing or *emm* typing (requires culture of the organism) - Laboratory (accredited pathology lab: yes/no) |

* Only for children who self-reported sore throat

**Enables linkage of laboratory data with participant, clinical and epidemiological data

ǂIf more than one specimen is taken, each specimen must have a unique ID number

## References

1. Shulman ST, Bisno AL, Clegg HW, et al. Clinical practice guideline for the diagnosis and management of group A streptococcal pharyngitis: 2012 update by the Infectious Diseases Society of America. *Clinical infectious diseases.* 2012;55(10):e86-e102.

2. Hysmith ND, Kaplan EL, Cleary PP, Johnson DR, Penfound TA, Dale JB. Prospective longitudinal analysis of immune responses in pediatric subjects after pharyngeal acquisition of group A streptococci. *Journal of the Pediatric Infectious Diseases Society.* 2017;6(2):187-196.

3. McDade TW, Williams S, Snodgrass JJ. What a drop can do: dried blood spots as a minimally invasive method for integrating biomarkers into population-based research. *Demography.* 2007;44(4):899-925.

4. Doshi RH, Alfonso VH, Hoff NA, et al. Evidence of mumps infection among children in the Democratic Republic of Congo. *The Pediatric infectious disease journal.* 2017;36(5):462-466.

5. Joseph J, Kent N, Bowen A, et al. Immuno-nephelometric determination of group streptococcal anti-streptolysin O titres (ASOT) from dried blood spots: Method for validating a new assay. *Journal of Immunological Methods.* 2017;448:59-65.

6. Bennett J, Moreland NJ, Oliver J, et al. Understanding group A streptococcal pharyngitis and skin infections as causes of rheumatic fever: protocol for a prospective disease incidence study. *BMC infectious diseases.* 2019;19(1):633.

7. Whitcombe AL, Han F, McAlister SM, et al. An eight-plex immunoassay for Group A streptococcus serology and vaccine development. *Journal of Immunological Methods.* 2022;500:113194.

8. Okello E, Ndagire E, Muhamed B, et al. Incidence of acute rheumatic fever in northern and western Uganda: a prospective, population-based study. *The Lancet Global Health.* 2021;9(10):e1423-e1430.

9. World Health Organization. Surveillance standards for vaccine-preventable diseases. Geneva: World Health Organization. 2018.

10. World Health Organization. A definition for community-based surveillance and a way forward: results of the WHO global technical meeting, France, 26 to 28 June 2018. *Eurosurveillance.* 2019;24(2).

11. Deutscher M, Van Beneden C, Burton D, et al. Putting surveillance data into context: the role of health care utilization surveys in understanding population burden of pneumonia in developing countries. *Journal of epidemiology and global health.* 2012;2(2):73-81.

12. U.S. Department of Health and Human Services. *E6(R2) Good Clinical Practice: Integrated Addendum to ICH E6(R1) Guidance for Industry* Maryland 2018.

13. Solares JRA, Raimondi FED, Zhu Y, et al. Deep learning for electronic health records: A comparative review of multiple deep neural architectures. *Journal of biomedical informatics.* 2020;101:103337.

14. Wang Z, Shah AD, Tate AR, Denaxas S, Shawe-Taylor J, Hemingway H. Extracting diagnoses and investigation results from unstructured text in electronic health records by semi-supervised machine learning. *PLoS One.* 2012;7(1):e30412.

15. Brodsky L. Modern assessment of tonsils and adenoids. *Pediatric Clinics of North America.* 1989;36(6):1551-1569.

1. a Note that specific antibody tests, such as SpnA (*Streptococcus pyogenes* nuclease A) serology, may be added to clinical practice as newer technologies become available. [↑](#footnote-ref-1)
2. Presence of Strep A after completion of treatment or ≥30 days after acute infection. [↑](#footnote-ref-2)
3. Point-of-care tests (POCTs) such as RADT and NAAT should only be used as part of a case definition in countries where they have been validated as a good test for the detection of Strep A in the oropharynx. RADTs have been shown to perform poorly relative to culture in many countries. Therefore, a positive POCT is an acceptable indicator of the presence of Strep A, but a negative POCT is a poor indicator of the absence of Strep A. [↑](#footnote-ref-3)
